# Early Empowerment Strategies Boost Self-Efficacy to Improve Cardiovascular Health Behaviors

**DOI:** 10.5539/gjhs.v8n9p322

**Published:** 2016-01-31

**Authors:** Mariam Kashani, Arn H Eliasson, Elaine M Walizer, Clarie E Fuller, Renata J Engler, Todd C Villines, Marina N Vernalis

**Affiliations:** 1 Integrative Cardiac Health Project, Walter Reed Military Medical Center, USA

**Keywords:** cardiovascular diseases, health behavior, lifestyle, prevention, risk factors, risk reduction, self-efficacy

## Abstract

**Background:**

Self-efficacy, defined as confidence in the ability to carry out behavior to achieve a desired goal, is considered to be a prerequisite for behavior change. Self-efficacy correlates with cardiovascular health although optimal timing to incorporate self-efficacy strategies is not well established. We sought to study the effect of an empowerment approach implemented in the introductory phase of a multicomponent lifestyle intervention on cardiovascular health outcomes.

**Design:**

Prospective intervention cohort study.

**Methods:**

Patients in the Integrative Cardiac Health Project Registry, a prospective lifestyle change program for the prevention of cardiovascular disease were analyzed for behavioral changes by survey, at baseline and one year, in the domains of nutrition, exercise, stress management and sleep. Self-efficacy questionnaires were administered at baseline and after the empowerment intervention, at 8 weeks.

**Results:**

Of 119 consecutive registry completers, 60 comprised a high self-efficacy group (scoring at or above the median of 36 points) and 59 the low self-efficacy group (scoring below median). Self-efficacy scores increased irrespective of baseline self-efficacy but the largest gains in self-efficacy occurred in patients who ranked in the lower half for self-efficacy at baseline. This lower self-efficacy group demonstrated behavioral gains that erased differences between the high and low self-efficacy groups.

**Conclusions:**

A boost to self-efficacy early in a lifestyle intervention program produces significant improvements in behavioral outcomes. Employing empowerment in an early phase may be a critical strategy to improve self-efficacy and lower risk in individuals vulnerable to cardiovascular disease.

## 1. Introduction

Cardiovascular disease (CVD) is the leading cause of death in Westernized nations (World Health Organization, 2010). Patients living with CVD experience decreased quality of life (Lewis et al., 2014), increase their utilization of health care resources (Tung et al., 1999) and decrease their economic productivity (Meland, Grønhaug, Oystese, & Mildestvedt, 2011). CVD prevention has therefore become a major goal of health care systems and medical professional societies (Eckel et al., 2014).

The main strategy in CVD prevention is to identify and improve risk factors (Eckel et al., 2014). Sustained improvements in CVD risk reduction requires that patients be made aware of their individual risk factors as well as their lifestyle behaviors that affect those risk factors such as avoiding tobacco use, making healthful nutrition choices, getting adequate exercise, managing stress levels, and getting adequate quantity and quality of sleep.

However, awareness alone is not adequate to change lifestyle behaviors affecting CVD risk (Elis et al., 2008), (Scotto, Waechter, & Rosneck, 2011). For behavior change, social-cognitive theory has proposed the concept of self-efficacy, defined as a person’s belief in his/her ability to carry out behavior to achieve a desired goal (Bandura, 1977). A number of studies have shown that self-efficacy is a prerequisite for making behavioral changes for the self-management of chronic conditions such as hypertension (Criswell, Weber, Xu, & Carter, 2010; Warren-Findlow, Seymour, & Brunner Huber, 2012), overweight (Linde, Rothman, Baldwin, Jeffery, 2006; Roach et al., 2003), and addictions (Kadden, & Litt, 2011).

Integral to a collaborative care model for chronic disease is patient empowerment, which is defined as helping patients to develop the inherent capacity to be responsible for one’s own life (Funnell, & Anderson, 2003). Empowerment approaches include interactive teaching strategies designed to involve patients in problem solving and as a result impact self-efficacy. Although studies support the utility of this approach, health professionals need a way to operationalize the empowerment of patients (Anderson, & Funnell, 2005). To lower CVD risk and improve adherence to healthy lifestyle change, strategies must be implemented to empower patients by enhancing self-efficacy.

There is an inverse relationship between self-efficacy and CVD risk factor profiles (Bailey, Kashani, Eliasson, & Vernalis, 2013), (Eliasson et al., 2015). However, the importance of self-efficacy for the management of CVD risks is not well established. Prior studies on this patient have shown mixed results. An observational study showed strong associations of high self-efficacy and adherence to two of four healthful behaviors for CVD (Sol, van der Graaf, van Peterson, & Visseren, 2011). A sub-analysis of a large prospective trial for treatment of hypertension showed that self-efficacy scores could not predict behavior change (Wingo et al., 2013). One randomized trial showed a lack of power for self-efficacy to predict adherence to 6 of 9 healthful behaviors (Sol, van der Graaf, van der Bijl, Goessens, & Visseren, 2008) and a second randomized trial showed equivocal results of an intervention to increase self-efficacy for exercise in cardiac rehabilitation patients (Barkley, & Fahrenwald, 2013). Little is known about the appropriate timing or mechanism for the implementation of self-efficacy enhancing strategies to achieve successful behavior change.

In the present study, we investigated the impact of an intervention designed to enhance self-efficacy by giving patients an early boost using an empowerment approach to improve adherence to healthy lifestyle behaviors. The empowerment intervention was implemented as part of a cardiovascular (CV) health program targeting behaviors in the areas of nutrition, exercise, perceived stress and sleep.

## 2. Methods

The Integrative Cardiac Health Project (ICHP) is a prospective registry of patients enrolling in a 12-month CV health program. The study has been registered with clinicaltrials.gov and may be found using identifier NCT01975181. All patients give informed consent for participation in the registry and the study is being conducted according to the principles stated in the Declaration of Helsinki.

Patients are self-referred or referred by a healthcare provider to assess their CVD risks and to learn how those risks can be improved through lifestyle behavior changes. Patients participating in ICHP are men and women over 17 years of age who are eligible for care in the Department of Defense Healthcare System. Participants are comprised of active duty service members, dependents of service members, and retirees from active service along with their dependents. As such, the participants in ICHP include both genders with a broad spectrum of ages, races and ethnicities. Some patients entering ICHP have diagnosed coronary heart disease but the large majority is seeking to reduce CV risk factors.

Upon entry to ICHP, patients meet with a nurse practitioner (NP) to undergo a CV-focused history and physical examination and submit a cardiac-relevant laboratory panel of tests. Based on this baseline assessment, patients are categorized as low, intermediate, or high risk for CVD by the Framingham Risk Score, the most widely used CVD risk estimator. Family history of premature CVD was collected and defined as a parent or sibling who had a CV event before the age of 55 in men and 65 in women. Patients also complete a series of validated questionnaires to determine their individual pattern of lifestyle behaviors.

Specific questionnaires focus on the domains of the program and are administered at baseline and at program completion, at 12 months: nutrition (Rate Your Plate), exercise (minutes of continuous exercise per week), stress (Perceived Stress Scale), and sleep (Pittsburgh Sleep Quality Index, Fatigue Visual-Analog Scale). A CV-relevant Self-Efficacy Questionnaire is administered at baseline and after an empowerment workshop, at 8 weeks from baseline (See Table 1).

**Table 1:** Time Points for Program Milestones

	Time 1 (Baseline)	Time 2 (8 Weeks)	Time 3 (12 Months)
**Framingham Risk Calculation**	X		
**Self-Efficacy Questionnaire**	X	X	
**Empowerment Intervention**	X	X	
**Rate-Your-Plate Nutrition Score**	X		X
**Exercise Minutes per Week**	X		X
**Perceived Stress Scale**	X		X
**Pittsburgh Sleep Quality Index**	X		X
**Fatigue Score**	X		X

**Self-Efficacy Questionnaire:** Self-efficacy was measured with the adapted diabetes mellitus type 2 self-efficacy scale. Since most self-management tasks apply generally to chronic diseases as a whole, this scale was used to measure the level of confidence people have about their ability to perform the self-management tasks necessary to reduce vascular risk. The 9-item questionnaire is scored on a 5-point Likert scale, with a higher self-efficacy score corresponding with better self-efficacy. Reliability of the questionnaire was tested with a Cronbach’s alpha of 0.69 (Sol, van der Graaf, van der Bijl, Goessens, & Visseren, 2006).

**Framingham Risk Score:** Cardiovascular disease risk was calculated using the standard FRS Hard Coronary Heart Disease (10-Year Risk) (National Cholesterol Education Program Expert Panel on Detection, Evaluation, and Treatment of High Blood Cholesterol in Adults, 2002). The tool, which currently forms the foundation of current primary prevention guidelines, uses age, gender, total cholesterol, HDL cholesterol, smoking history and blood pressure (BP) to calculate the risk of coronary heart disease outcomes (MI and coronary death) over the subsequent 10 years. Scores are categorized as low (<10), medium (11-19) and high (>20).

**Rate Your Plate (RYP):** This ICHP-modified 26-item nutrition questionnaire, originally developed in 1983 by the Pawtucket Heart Health Program (PHHP), consists of questions focusing on foods that contribute the most fat, saturated fat, and cholesterol to the American nutrition. In a calibration study, the RYP was compared with the widely used Willet food frequency questionnaire (FFQ). When the RYP was administered prior to the Willet FFQ, Pearson product-moment correlations ranged between -0.45 and -0.65 on fat variables and nutrition cholesterol (p < .001 for all correlations), thus having the capacity to quantitatively reflect intake of fat and saturated fat (Gans, Hixson, Eaton, & Lasater, 2000), (Gans et al., 1993). The RYP individual score can indicate whether the participant’s typical eating pattern is relatively high or low in fat, saturated fat and cholesterol. This questionnaire has been modified over the years to reflect changing national nutrition recommendations, fat-reduced foods now available in the marketplace, eating out and consideration of trans fatty acids in recommendations for spreads and cooking oils including the ICHP modifications to reflect use of beer, wine, alcohol, soda and other sugary drinks. Scores range from 26-78 with 26-42 reflecting least “heart healthy”; 43-60 middle ground, and; 61-78 most “heart healthy”.

**Perceived Stress Scale:** PSS-14 developed in 1983 (Cohen et al., 1983) is one of the most widely accepted of measurements of stress (Cohen, Kamarck, & Mermelstein, 1983). Validation studies show that the PSS-14 has an internal consistency reliability of 0.85 by Cronbach alpha and a test-retest reliability of 0.85. This 14-item questionnaire asks the patient how often certain experiences of stress occurred in the last month and is designed to measure the degree to which situations in one’s life are appraised as stressful. With item responses from 0 to 4, the range of possible scores is 0 to 56 with higher scores correlating with higher stress. The PSS is designed for use with community samples with at least a junior high school education. The items are easy to understand and the response alternatives are simple to grasp. Moreover, the questions are quite general in nature and hence relatively free of content specific to any subpopulation group. Scores in the low 20’s reveal moderate stress levels while scores approaching 30 are substantial and concerning.

**Pittsburgh Sleep Quality Index:** PSQI is a self-rated questionnaire used to assess sleep quality and disturbances over a 1-month time interval (Buysse, Reynolds 3rd, Monk, Berman, & Kupfer, 1989). Nineteen individual items generate seven component scores whose sum yields one global score with a range of 0 to 21. The psychometric and clinical properties of the PSQI suggest its utility both in clinical practice and research activities. A PSQI score greater than 5 has a diagnostic sensitivity of 89.6% and specificity of 86.5% (kappa = 0.75, *p* ≤ 0.001). Essentially, a global score of greater than 5 indicates a poor sleeper. Sleep perturbations can be categorized by scores as follows: 0 to 5 is a good sleep score; 6 to 10 shows mild sleep difficulty; 11 to 15 moderate sleep difficulty, and 16 to 21 severe sleeping difficulty.

**Fatigue Visual Analog Scale:** This scale is borrowed from the Stanford Patient Education Research Center where it was tested on 122 patients, with mean value of 4.89 and standard deviations of 2.71 (Stanford Patient Education Research Center). This fatigue scale asks patients to express their experience of fatigue from 0 to 10 for the previous 2 week period. Patients who circle 5 to 6 express mild fatigue, 7 to 8 moderate fatigue, and 9 to 10 severe fatigue.

**Empowerment Intervention:** Empowerment strategies for patients are comprised of a comprehensive risk assessment report with detailed lifestyle recommendations for optimizing risk reduction generated by the NP. After two initial NP appointments which allow patients to problem-solve barriers to lifestyle change, clarify goals and identify motivations, patients attend a multi-disciplinary educational workshop. The workshop is comprised of an interactive healthy food demonstration and stress management experience and focus on the impact of actionable behaviors on health. For the remainder of the program and after the workshop, patients receive ongoing motivational advice by health coaches in order to achieve healthy goals in the program’s domains as established by the NP in the first two weeks.

**Statistics:** Sample size calculation indicated that a sample of 100 participants would provide a stabilized and generalizable set of data. Plans were made to enroll approximately 115 patients to allow for a 15 percent rate of drop-out for failure to complete surveys properly. Data are presented as means with standard deviations or proportions. A median score was used to split patient Self-Efficacy Scores into two groups of nearly equal size allowing comparisons between high and low scoring groups. Demographic and survey health variables were compared using chi-square test for categorical variables, and Student’s t-test for continuous variables. Pearson product-moment correlation coefficients were used to measure correlations because the data sets were normally distributed as seen on inspection of population histograms. All tests assumed p < 0.05 as statistical significance.

## 3. Results

The study population is comprised of 119 consecutive graduates of the ICHP CV program. The demographic variables of the group are provided in Table 2. The population is generally late middle aged, evenly split between men and women, representative of a variety of races, predominantly married and living in a family unit. Patients had an average of 2.5 CVD risk factors each, thus comorbid illnesses were common. Of the 119 patients, 9 (8%) were diagnosed with coronary heart disease, 67 (66%) with dyslipidemia, 61 (51%) with hypertension, 39 (33%) with obstructive sleep apnea, 32 (27%) with depression, 9 (8%) with diabetes, and 25 (21%) with pre-diabetes. Family history of premature CVD was reported by 53 patients (45%) in the total group. There were 20 (17%) patients who served as caregivers for other family members at home.

**Table 2:** Demographic variables for participants in the ICHP Registry

		All Patients (n = 119)	Low Self-Efficacy* (n = 59)	High Self-Efficacy** (n = 60)	p value***
**Age (Years ± SD)**		56.5 ± 13.1	55.2 ± 13.7	57.8 ± 12.6	0.28

**Sex# men (%)**		57 (48)	28 (47)	29 (48)	0.92

**Race**	White	85	41	44	0.85
	Black	21	12	9	
	Hispanic	5	3	2	
	Asian	2	1	1	
	Other	6	2	4	

**Marital Status**	Single	10	8	2	0.18
	Married	94	45	49	
	Divorced	11	5	6	
	Separated	4	1	3	

**Number of Children**	One	25	14	11	0.54
	Two	13	6	7	
	Three	39	18	21	
	Four or More	8	2	6	

* Low Self-Efficacy is defined as the group scoring below the median score of 36 points.

** High Self-Efficacy is defined as the group scoring at or above the median score of 36 points.

*** p value denotes statistical difference between Low and High Self-Efficacy Groups by t-test for age and by chi square test for other variables.

Inspection of a histogram of the self-efficacy scores revealed that these were normally distributed. The median score (36 points) of the self-efficacy questionnaire measured at entry to ICHP was used to divide the participants into high (n=60) and low scorers (n=59). Demographic variables were not different for high and low self-efficacy subgroups (See Table 2). Framingham risk scores were calculated for each patient showing that nearly one third of patients were at intermediate or high risk for a CVD event over 10 years. Low self-efficacy patients were at higher CVD risk than high self-efficacy patients by Framingham estimation (See Table 3).

**Table 3:** At baseline, low Self-Efficacy correlates with higher CVD risk

	Medium or High Risk by Framingham	p value*
Low Self-Efficacy (**n=59)**	36%	0.04
High Self-Efficacy (**n=60)**	22%	

* Chi square analysis shows a significant difference between groups.

At baseline, the low self-efficacy group entered the ICHP program with lower scores for a healthy nutrition, less exercise minutes per week, higher levels of perceived stress, poorer sleep quality and greater fatigue (See Table 4).

**Table 4:** Change in Outcomes from Baseline to Completion According to Self-Efficacy Score

		All Patients (n = 119)	Low Self-Efficacy* (n = 59)	High Self-Efficacy** (n = 60)	p value***
**Self-Efficacy (of 45 points)**	Baseline	34.5 ± 6.5	29.1 ± 0.8	39.9 ± 3.0	NA
	Completion	40.3 ± 4.2	38.2 ± 4.6	42.4 ± 2.2	< 0.001
	Change	5.8, p<0.001	9.1, p<0.001	2.5, p<0.001	< 0.001

**Nutrition (of 78 points)**	Baseline	61.7 ± 8.3	58.9 ± 4.1	64.5 ± 7.4	< 0.001
	Completion	67.1 ± 6.0	65.7 ± 6.6	68.6 ± 5.0	0.008
	Change	5.4, p<0.001	6.8, p<0.001	4.1, p<0.001	0.01

**Exercise (minutes per week)**	Baseline	156 ± 125	110 ± 87	201 ± 141	< 0.001
	Completion	220 ± 163	186 ± 157	253 ± 163	0.02
	Change	64, p<0.001	76, p=0.002	52, p=0.06	0.16

**Perceived Stress (of 56 points)**	Baseline	20.1 ± 9.1	22.0 ± 8.5	18.3 ± 9.3	0.02
	Completion	17.2 ± 8.6	18.3 ± 8.7	16.1 ± 8.4	0.16
	Change	2.9, p=0.01	3.7, p=0.02	2.2, p=0.18	0.18

**Sleep Quality (of 21 points)**	Baseline	7.1 ± 3.9	7.9 ± 4.3	6.2 ± 3.2	0.02
	Completion	4.7 ± 3.5	5.3 ± 4.1	4.1 ± 2.7	0.06
	Change	2.4, p<0.001	2.6, p=0.001	2.1, p<0.001	0.53

**Fatigue (of 10 points)**	Baseline	4.3 ± 2.5	5.0 ± 2.4	3.6 ± 2.3	0.001
	Completion	3.0 ± 2.2	3.2 ± 2.3	2.9 ± 2.1	0.39
	Change	1.3, p<0.001	1.8, p<0.001	0.7, p=0.07	0.01

* Low Self-Efficacy is defined as the group scoring below the median score of 36 points.

** High Self-Efficacy is defined as the group scoring at or above the median score of 36 points.

*** p value denotes statistical difference by t-test between Low and High Self-Efficacy Groups.

These findings were corroborated with Pearson r product-moment correlations which showed a strong correlation of nutrition scores and moderately strong correlations of exercise minutes, lower stress scores, and better sleep quality with total self-efficacy scores (See Table 5).

**Table 5:** At baseline, improvements in Self-Efficacy Score correlate with improvements in health indices.

	Nutrition Score	Exercise Minutes	Stress Levels	Sleep Quality
**Total SE Score**	0.47 (p<0.001)	0.37 (p<0.001)	0.30 (p=0.03)	0.36 (p<0.001)

The Pearson r coefficients show a strong correlation between baseline self-efficacy score and nutrition score and moderately strong correlations for exercise, stress and sleep.

In response to the empowerment intervention of the ICHP program, 98 of the total 119 patients (82%) showed gains in self-efficacy with an average improvement of 7.2 ± 4.4 points; 11 (9%) showed no change; and 10 (8%) decreased their self-efficacy an average of 2.0 ± 1.2 points. In the group of 59 patients with low self-efficacy at program entry, 58 (98%) showed improvements averaging 9.4 ± 4.4 points in self-efficacy and only one (2%) decreased self-efficacy by 2.0 points.

Among all 119 participants, only three (3%) were active tobacco smokers, each reporting current smoking of 2 cigarettes per day. Nineteen other patients were former smokers, having an average 1 pack per day history of smoking, and having quit an average of 26 years prior. Since tobacco use occurred so infrequently in the study population, further analysis of tobacco use was not performed.

## 4. Discussion

The salient finding of the current study is that a boost to self-efficacy early in a lifestyle intervention program produces substantial improvements in behavioral outcomes. The overwhelming majority of patients responded with improved self-efficacy scores. Self-efficacy scores increased irrespective of baseline self-efficacy.

Though patients in both the low and the high self-efficacy groups showed improvements in self-efficacy and behavioral survey scores, the largest gains in self-efficacy occurred in patients who ranked in the lower half for self-efficacy at baseline. This lower self-efficacy group also demonstrated behavioral improvements that erased differences between the high and low self-efficacy groups or at least provided a substantial “catch-up” such that the scores on completion of the program were approaching or better than baseline scores for the high self-efficacy group.

Our findings agree with prior reports that self-efficacy scores at baseline correlate with the cardiovascular risk profile. Indeed in our population, patients with low self-efficacy scores were found to have a higher predicted cardiovascular risk by Framingham Risk Score in addition to less healthy cardiovascular behaviors (Table 3). Given the burden of CV risk and comorbid illness in the low self-efficacy group it is critical to provide self-care behavioral tools to overcome lifestyle behavioral change barriers.

Prior studies assessing the impact of self-efficacy on adherence to behavior change have frequently been limited to a single behavioral dimension such as nutrition (Sharp, & Salyer, 2012), (Timlin, Shores, & Reicks, 2002), (Nothwehr, 2008), (Cha, 2014) or exercise (Slovinec D’Angelo, Pelletier, Reid, & Huta, 2014), (Schwarzer, Luszczynska, Ziegelmann, Scholz & Lippke, 2008). Investigations evaluating the impact of a self-efficacy intervention on multiple behaviors have had mixed results. One randomized controlled trial reported positive effects on nutrition and exercise but not on smoking and alcohol intake (Sol et al., 2008). Another prospective cohort study showed the beneficial effect of increased self-efficacy on nutrition, exercise and stress management (Clark, & Dodge, 1999). The present prospective cohort study shows positive effects of improved self-efficacy on nutrition, exercise, and stress management behaviors but extends the positive effects to sleep improvement as well.

Employing an empowerment intervention early in the sequence of events in a heart-healthy program provides a mechanism for increased patient self-efficacy. Our findings validate numerous studies showing that interventions that aim to empower patients are valuable in promoting patient well-being, decision-making and self-management of chronic disease (Aujoulat, Marcolongo, Bonadiman, & Deccache, 2008).

The results of the current study appear to be generalizable to other locations and institutions. This group of patients at risk for CVD mirrors the population at large with regard to demographic profile and comorbid illnesses. The intervention that was provided does not require special equipment or resources and is therefore scalable and could be duplicated in other centers targeting CVD prevention.

The current study has limitations. Because the intervention aimed at multiple dimensions of healthy CV behaviors, it is not possible to determine which aspects of the empowerment intervention were most effective. Likewise, with the current study design it is not possible to determine whether or not there was a synergistic impact from improvement in one behavior that helped stimulate improvements in other behaviors. A second limitation is the use of measurement tools that rely on self-report. While these tools are validated instruments to measure the behaviors for which they were targeted, the use of objective measures would give more robust information and therefore be more convincing. Unfortunately, objective measures are complex (nutrition measures), unwieldy (actigraphy for exercise and sleep), or do not readily exist (stress management).

In summary, the results of this study support the idea that a lifestyle behavioral change program aimed at providing an early boost to self-efficacy is feasible and can yield positive results. These findings are particularly significant in high-risk patients who are vulnerable to CVD and may be in a position to make critical behavioral lifestyle modifications to lower their risk of overt disease. Further study is warranted to measure the impact that such behavior changes have on prevention of CVD events.
